# Childhood Pneumonia Screener: a concept

**DOI:** 10.15172/pneu.2014.5/515

**Published:** 2014-12-01

**Authors:** Jukka Räsänen, Noam Gavriely

**Affiliations:** 17 Department of Anesthesiology, H. Lee Moffitt Cancer Center, 12902 USF Magnolia Drive, Tampa, FL 33612 USA; 27 Karmel Medical, Haifa, Israel

**Keywords:** pneumonia, acoustics, global health, telemedicine, childhood mortality

## Abstract

Childhood pneumonia continues to be the number one cause of death in children under five years of age in developing countries. In addition to mortality, pneumonia constitutes an enormous economic and social burden because late diagnosis is associated with high cost of treatment and often leads to chronic health problems. There are several bottlenecks in developing countries in the case flow of a child with lung infection: 1) recognising the symptoms as a reason to seek care, 2) getting the patient to a first-tier health facility, 3) scarcity of trained healthcare personnel who can diagnose the condition and its severity, 4) access to a second-tier facility in severe cases. These factors are commonly present in rural areas but even in more urban settings, access to a physician is often delayed. The Childhood Pneumonia Screener project aims at bridging the diagnostic gap using emerging technology. Mobile “smart” phone communication with several inexpensive dedicated sensors is proposed as a rapid data-collection and transmission unit that is connected to a central location where trained personnel assisted by sophisticated signal processing algorithms, evaluate the data and determine if the child is likely to have pneumonia and what the level and urgency of care should be.

## 1. Introduction

Childhood pneumonia is widely recognised as the leading cause of mortality globally among children less than five years of age; representing 18% of the deaths in that age group [1,2]. The percentage translates into 1,396,000 deaths annually (2010), of which approximately one fifth are neonates [1]. Over 99% of these deaths occur in developing countries; 85% in Sub-Saharan Africa and South Asia combined, and 39% in the five least developed countries (India, Nigeria, Democratic Republic of the Congo, Pakistan, and Ethiopia). The factors predisposing to childhood pneumonia in developing countries are well known: poor nutrition of mothers and children leading to low birth weight, lack of breastfeeding, indoor air pollution, poor hygiene and crowding, comorbidities, and absence of common preventive measures such as vaccination. In most cases, a cure does exist if it can be administered in time [3,4].

Administration of antibiotics to a child with suspected pneumonia is known to be lifesaving but before treatment can begin, the child’s caregiver must first understand that the child is seriously ill. Two thirds of caregivers in developed countries, but less than half in the most underserved areas, will recognise difficult breathing or rapid respiratory rate as a reason to seek care [1]. Even if these symptoms are recognised, the illness may go undetected for a time because the tasks of daily life consume much of the attention of the adult caregivers. If help is sought, the logistics of reaching a health facility may be adversely affected by terrain, distance, and lack of transportation. Once the patient arrives at the health facility, the nature of the condition and its severity must be correctly identified by the staff. Suspicion of pneumonia by healthcare workers, according to the current World Health Organization Integrated Management of Childhood Illness guidelines [5,6], rests on the detection of rapid respiratory rate and/or chest indrawing which will trigger the administration of antibiotics. Expertise and equipment to facilitate a more refined diagnosis or to identify children who might require more advanced care, such as oxygen and parenteral antibiotics, is rarely available.

The lack of specific diagnosis of childhood pneumonia likely results in both unnecessary administration of antibiotics and, more importantly, failure of healthcare workers to recognise the severity of a child’s condition. As important as diagnosing and triaging an existing pneumonia, is turning the focus on some other reason for the child’s symptoms when lung infection is ruled out: A false positive diagnosis of lung infection may detract from finding the true reason for the child’s symptoms and result in erroneous therapy when a course of antibiotics is not the proper treatment.

The Childhood Pneumonia Screener (CPS) project aims to provide a simple tool to improve the accuracy of the first-stage healthcare provider in diagnosing pneumonia. The CPS provides a recording of heart rate, oxyhaemoglobin saturation, and body temperature with systematic auscultation which, compared to a measurement of respiratory rate alone, would be expected to improve the basis for decision making in these cases [7–10]. Additionally, the benefits of systematic physical examinations and electronic documentation of the findings go beyond the individual patient because they facilitate the understanding of the epidemiology of respiratory illness in a given area and facilitate timely interventions should increasing numbers or clusters of cases be detected.

## 2. The Childhood Pneumonia Screener (CPS)

### 2.1 System Requirements

The CPS requires the availability of cellular communication infrastructure that can support mobile “smart” phones. Importantly, this support includes the need for electrical power to charge the phone where the front-end units are used. The communication continuity should be sufficient to be trusted as a component in a critical medical decision-making system. In addition, the following high-level performance requirements have been identified: 1) The CPS should provide a binary result to indicate whether a child does or does not have pneumonia. 2) When the CPS detects pneumonia, it should also provide a simple severity score. 3) The front-end device should be sufficiently simple to be operated by a minimally trained technician/care-giver. 4) The CPS data collection should take no more than 12 minutes to complete. 5) The central analysis and data review should take no more than 15 minutes to complete once the data has been received. 6) The central location personnel should be trained to use the system, interpret the analysis output, recognise visual signs of respiratory distress, and evaluate the questionnaire input. They also must have language and communication skills to handle emergencies from a remote location. 7) The technology and its operation should be affordable.

### 2.2 The CPS high-level concept and system components

The CPS consists of two main elements: 1) the front-end field unit; and 2) the central signal processing, analysis and interpretation unit. The front-end data-gathering device will have effective communication capacity to the central or regional location where the bulk of the analysis is performed. An important concept of the proposed project is that centrally located trained healthcare personnel will be the ultimate pivot of the data interpretation and decisionmaking. No closed-loop or automated decision-making is contemplated within the scope of this project even though it may be feasible to expand the project in that direction later. Additional high-level principles to be implemented include 1) minimal front-end point-of-care data processing to determine acceptability of signal quality, 2) multimodal sensing capability, 3) icon-based graphic guidance and prompting, and 4) real-time or off-line remote human verification and decision-making.

#### 2.2.1 The CPS front-end field unit

**Figure 1:**
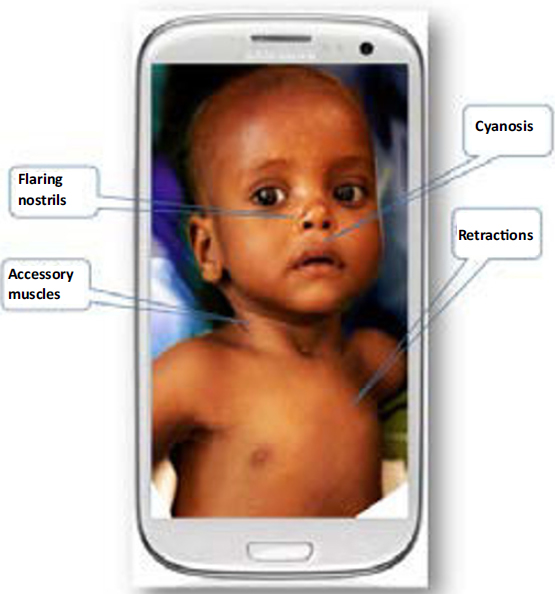
Screenshot of proposed video image of a patient on the Childhood Pneumonia Screener field unit.

**Figure 2:**
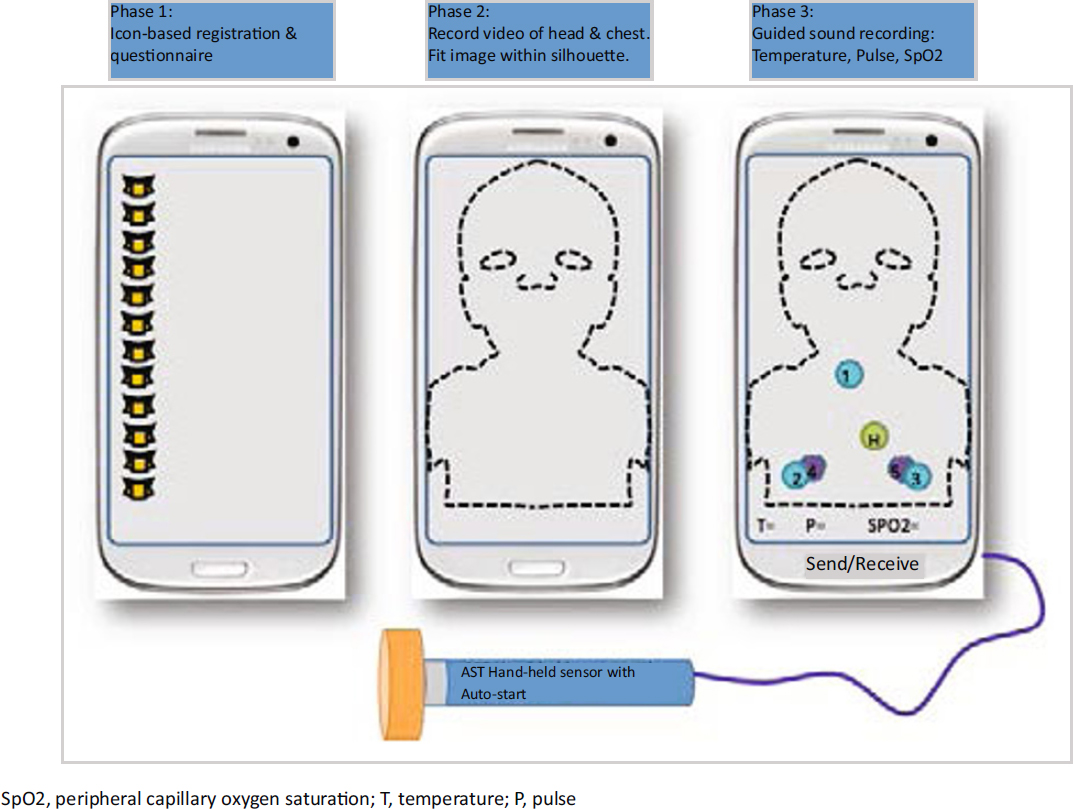
Diagram of the Childhood Pneumonia Screener field unit at three phases of data collection. Note that auscultation point 1 is over the trachea or manubrium, points 2 and 3 are on the anterior chest wall, points 4 and 5 are on the posterior bases and point H is on the left sternal border for heart sounds.

The front-end field unit consists of a mobile “smart” phone that is capable of recording and transmitting data, photographs, audio and video clips using a structured application that guides the data collection (Figure 1). The application consists of a step-by-step process starting from data entry on the child’s identification including address (when available) and mode of communication with caregiver, date of birth, weight, height, and indication of previous evaluation by the CPS system. The CPS will then present a questionnaire seeking information on the present illness, relevant previous health history, illnesses in the immediate surrounding family, and current medications. Next, the CPS will guide the user to record a 15-second frontal view of the head and thorax of the exposed child. The data-collection application will verify locally that the image fulfills certain quality criteria of lighting, fit within the frame and focus. The next step will consist of recording lung and heart sounds from the patient’s chest. The application will prompt the user to place the hand-held acoustic sensor (Figure 2) on specific locations for a 15-second recording at each location. A built in mechanism will determine that the sensor is held properly with appropriate contact pressure and will activate the recording only when the sensor is in touch with the body. An ambient microphone will be used to monitor environmental noise. The application will issue a warning if the noise level is too high. A pulse oximeter will then be used to record the haemoglobin oxygen saturation and heart rate. Failure to obtain a stable oximetry reading will flag a high risk for severity.

**Figure 3:**
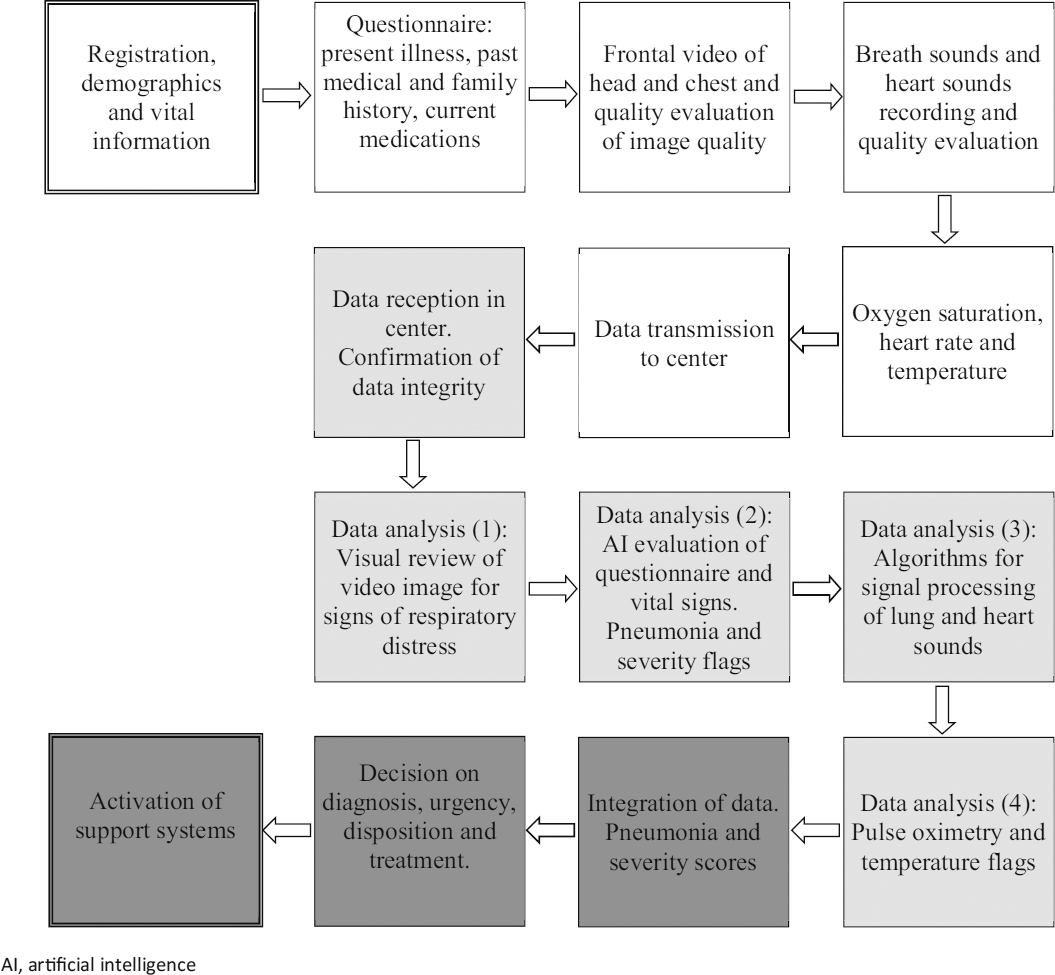
Flowchart of the data collection and analysis processes of the Childhood Pneumonia Screener.

The data will be transmitted to the central location under supervision of the application and a copy of the data will be kept in the phone’s memory as needed. A flowchart of the data acquisition process is shown in Figure 3. Commercially available and proprietary components needed for the field unit are listed in Table 1.

**Table 1:** Commercially available (to be bought) and proprietary (to be manufactured) components of the Childhood Pneumonia Screener field unit.

Commercially available components	Proprietary components
The mobile “smart” phone with built-in video camera and microphone	Software application for guiding data collection, quality evaluation and transmission of data
Lung and heart sound sensor	Archival of collected data (back-up)
Pulse oximeter	
Thermometer	

#### 2.2.2 Processing of the data in the central hub

The processing of the data in the central location consists of two main elements: 1) an “artificial intelligence” set of software algorithms to analyse the incoming data; and 2) a trained person to observe the video image, interpret the information coming from the algorithm, integrate the information to decide on a pneumonia diagnosis and severity score, and decide on patient disposition and treatment. The main requirements of the central hub include 1) personnel and their training, 2) communication technology, 3) computation technology loaded with signal processing software, 4) integration augmentation tools (scores), 5) archival of data, and 6) follow-up.

A unique component of the central hub processing capability is an automated signal processing set of algorithms of lung and heart sounds. The algorithms will analyse the lung sounds to determine respiratory rate and breathing waveform, detect the presence of wheezes or crackles and their timing within the respiratory cycle. The algorithms will also be used to assess heart sounds by measuring heart rate, systole/diastole ratio, heart rate variability, and S1 amplitude variability (a measure of respiratory effort, similar to pulsus paradoxus). The algorithms are capable of removing, to a certain degree, the ambient noise interference. Another algorithm will be developed to analyse the questionnaire information with specific indicators for the presence or absence of pneumonia, and if one is present, its severity. Finally, the formatted integration of all data will be presented to the healthcare professional manning the central hub so that an expedited decision can be made.

## 3. Working with existing healthcare providers and authorities

In developing countries healthcare access points vary from region to region. Appropriate first step caregivers who would be expected to use the detection device include community health workers, personnel at health centers or posts, and local physicians. These providers may also be able to start first line therapy when instructed to do so.

A second-tier provider needs to be identified for each region to accept a patient whose condition is determined unstable or poorly responsive to first line treatment. The second-tier provider needs to be accessible to the patient and within reasonable transport distance considering the child’s illness. The evaluation and care provided at the second-tier needs to be within the financial reach of the patient or community.

A tertiary level of care will be set up and equipped to receive the data transmitted by the device and to provide the automated and human decision support regarding the likelihood of pneumonia, the seriousness of the patient’s condition, and the management plan. This tertiary level is ideally a hospital with a physician or nurse practitioner experienced in the triage and treatment of children and childhood pneumonia, and knowledgeable of the conditions and facilities local to the patients that are being served. The tertiary facility need not be in the vicinity of the patient as long as a data connection can be established.

It is also necessary to ascertain that the supporting data transfer networks exist and are functional in the area, and that the receiving facility is equipped and staffed appropriately. Cellular network access even in developing countries can be surprisingly good, certainly in the urban areas and along the main roads. In the data receiving facility, automated decision support systems can eventually be built to respond to triage requests at times when human coverage cannot be assured, with human backup from other locations.

For the Childhood Pneumonia Screener project to be successful, it is essential to work with the local healthcare structure to secure a chain of case management that brings the patient to the healthcare access point in time. Of the several barriers that exist in developing countries for establishing an effective case management flow for an obviously ill child, this project addresses one, namely improved diagnosis and triage of pneumonia. While the problems of recognising illness in a child and understanding that cure is possible could be addressed with education, the logistic difficulties of bringing the patient to a healthcare access point may be overwhelming in some locations. On the other hand, there are many large concentrations of underserved children in which conditions do allow development of timely case flow and where proper pneumonia screening would be of help.

## 4. Verification and validation

After completion of laboratory tests with recorded signals, the CPS system will be evaluated in three phases.

Clinical evaluation of the system within a controlled environment such as an emergency room in a developed country will constitute the first phase. The system’s output and user triage decisions will be compared to those of the treating physicians. The system’s parameters will then be adjusted with the aim to reach a very high (>95%) sensitivity for detection of severe pneumonia. The false positive rate will be evaluated with the aim to achieve better than 75% specificity. This means that for every four “positive” detections, three will correctly indicate a severe pneumonia and one will occur in a patient who does not actually have the condition. This weighted receiver operator characteristic (ROC) skewness is based on balancing the consequences of missing a real case as opposed to over-diagnosing one. Any feedback and issues raised by this preliminary evaluation will be the basis for a second phase refinement of the system and its algorithms.

The second phase of evaluation will be a real-life test of the system in a single center with up to 25 field units. This phase will incorporate and evaluate the training protocols for the personnel using the system, the communications infrastructure and the chain of patient management. Feedback from this phase will again be used to refine the system and its components. The data from the second phase of evaluation will be used to determine the power required for the third phase in order to evaluate secondary outcomes.

The third phase of evaluation will consist of an outcome study reaching a population of about 100,000 children in potential need of the system. The desired outcome is a significant drop in childhood mortality due to severe pneumonia.

## 5. Critical evaluation and risk analysis

There is a range of potential shortcomings in remote diagnosis of pneumonia and the assessment of its severity. The key to mitigating this risk is the incorporation of a centrally located trained healthcare person in the loop. The visual inspection of the video and the unstructured phone communication with the operator of the field unit will prevent mere reliance on technical data and signal processing algorithms. The system is inherently lacking the capability to perform a chest radiograph or measure blood gases and blood pressure; otherwise, it contains all the essential components of history taking, inspection, auscultation and vital signs. The evidence necessitating a chest radiograph at the time of the initial triage of childhood pneumonia is inconclusive in the paediatric literature [11,12]. In any event, this is essentially an academic issue because in the locations where the CPS is to be used, it is not feasible to take a chest radiograph of each child suspected of pneumonia and to have it interpreted in a reasonable timeframe by a competent radiologist. The fact that a chest radiograph may be negative around the onset of pneumonia is also well known [11–13].

Other potential risks are associated with the conditions of data acquisition. The field user may or may not be able to communicate effectively with the parent, or may misinterpret their descriptions of the child’s illness when entering the data into the questionnaire. Ambient noise may interfere with the acoustic data quality and obtaining video and a pulse oximetry reading of a child who cannot be calmed down may be challenging. Communication infrastructure failures are to be anticipated and weighed into the overall risk-analysis.

The algorithms used to analyse the sounds and extract the features such as presence of crackles and wheezes, heart rate and respiratory rate from the sensor data are all based on statistical parameters and are calibrated against human observers who are inherently imprecise.

The highest risk and limitation is the lack of buy-in on part of the healthcare authority and the target population. It is essential to work closely and early on with people who are intimately familiar with the mentality and cultural traditions of the people who may benefit from the system. A properly powered outcome study with a well-conceived control group is key to the success of the incorporation process.

## References

[1] UNICEF. (2012). Pneumonia and Diarrhoea-tackling the deadliest diseases for the world’s poorest children.

[2] Izadnegahdar R, Cohen AL, Klugman KP, Qazi SA (2013). Childhood pneumonia in developing countries. Lancet Respir Med.

[3] Ayieko P, English M (2007). Case management of childhood pneumonia in developing countries. Pediatr Infect Dis J.

[4] Sazawal S, Black RE (2003). Pneumonia Case Management Trials Group. Effect of pneumonia case management on mortality in neonates, infants, and preschool children: a meta-analysis of community-based trials. Lancet Infect Dis.

[5] World Health Organization. (2014). Revised WHO classification and treatment of childhood pneumonia at health facilities. Evidence summaries.

[6] World Health Organization. (2014). Revised WHO classification and treatment of childhood pneumonia at health facilities. Quick reference guide.

[7] Shah S, Bachur R, Kim D, Neuman MI (2010). Lack of predictive value of tachypnea in the diagnosis of pneumonia in children. Pediatr Infect Dis J.

[8] Neuman MI, Monuteaux MC, Scully KJ, Bachur RG (2011). Prediction of pneumonia in a pediatric emergency department. Pediatrics.

[9] Jadavji T, Law B, Lebel MH, Kennedy WA, Gold R, Wang EE (1997). A practical guide for the diagnosis and treatment of pediatric pneumonia. CMAJ.

[10] Bilkis MD, Gorgal N, Carbone M, Vazquez M, Albanese P, Branda MC (2010). Validation and development of a clinical prediction rule in clinically suspected communityacquired pneumonia. Pediatr Emerg Care.

[11] Bhutta ZA (2007). Dealing with childhood pneumonia in developing countries: how can we make a difference?. Arch Dis Child.

[12] Hazir T, Nisar YB, Qazi SA, Khan SF, Raza M, Zameer S (2006). Chest radiography in children aged 2–59 months diagnosed with non-severe pneumonia as defined by World Health Organization: descriptive multicentre study in Pakistan. BMJ.

[13] Hagaman JT, Rouan GW, Shipley RT, Panos RJ (2009). Admission chest radiograph lacks sensitivity in the diagnosis of community-acquired pneumonia. Am J Med Sci.

